# A Functional *NQO1* 609C>T Polymorphism and Risk of Gastrointestinal Cancers: A Meta-Analysis

**DOI:** 10.1371/journal.pone.0030566

**Published:** 2012-01-17

**Authors:** Hongping Yu, Hongliang Liu, Li-E Wang, Qingyi Wei

**Affiliations:** Department of Epidemiology, The University of Texas, M. D. Anderson Cancer Center, Houston, Texas, United States of America; Ohio State University Medical Center, United States of America

## Abstract

**Background:**

The functional polymorphism (rs1800566) in the *NQO1* gene, a 609C>T substitution, leading to proline-to-serine amino-acid and enzyme activity changes, has been implicated in cancer risk, but individually published studies showed inconclusive results.

**Methodology/Principal Findings:**

We performed a meta-analysis of 20 publications with a total of 5,491 cases and 5,917 controls, mainly on gastrointestinal (GI) cancers. We summarized the data on the association between the *NQO1* 609C>T polymorphism and risk of GI cancers and performed subgroup analyses by ethnicity, cancer site, and study quality. We found that the variant CT heterozygous and CT/TT genotypes of the *NQO1* 609 C>T polymorphism were associated with a modestly increased risk of GI cancers (CT *vs.* CC: OR = 1.10, 95% CI = 1.01 – 1.19, *P*_heterogeneity_ = 0.27, *I* ^2^ = 0.15; CT/TT *vs.* CC: OR = 1.11, 95%CI = 1.02 – 1.20, *P*_heterogeneity_ = 0.14; *I* ^2^ = 0.27). Following further stratified analyses, the increased risk was only observed in subgroups of Caucasians, colorectal cancer in Caucasians, and high quality studies.

**Conclusions:**

This meta-analysis suggests that the *NQO1* 609T allele is a low-penetrance risk factor for GI cancers. Although the effect on GI cancers may be modified by ethnicity and cancer sites, small sample seizes of the subgroup analyses suggest that further larger studies are needed, especially for non-colorectal GI cancers in Caucasians and GI cancers in Asians.

## Introduction

Gastrointestinal (GI) cancers are the common malignant tumors in the world [1], [2], of which colorectal cancer is the third most common cancer in males and the second in females, with over 1.2 millions of new cases and 608,700 deaths occurred in 2008 [2]. It was estimated that cancers of the esophagus, stomach, colorectum, and liver accounted for 26.4% (3.4 millions) of the total new cancer cases and 32.8% (2.5 millions) of the total cancer deaths in 2008 worldwide [2]. Although the causes of these cancers are complex and heterogeneous, chronic inflammation, cigarette smoking, heavy alcohol drinking, and poor dietary pattern are generally considered possible risk factors for these cancers [3], [4], [5], [6], [7], [8], [9], [10], [11], [12], [13]. In addition, numerous case-control, family-based and twin studies have shown that inherited genetic factors have played an important role in susceptibility to these diseases [12], [13], [14], [15], [16], [17]. Recent genome-wide association studies have also identified some susceptible loci harboring common single nucleotide polymorphisms (SNPs) for risk of GI cancers, suggesting that the low-penetrance genes are also involved in the etiology of these diseases [18], [19], [20], [21], [22].

NAD(P)H:quinone oxidoreductase 1 (NQO1) is an obligate two-electron reductase, which reduces reactive quinones to less reactive and less toxic hydroquinones. The quinones are mainly derived from endogenous quinones, such as vitamin E quinone and ubiquinone, and exogenous quinones, such as exhaust gas, cigarette smoke or diet [23], [24]. This two-electron reduction prevents the formation of semiquinones and highly reactive oxygen species (ROS), thus protecting cells against oxidative stress, cytotoxicity, and mutagenicity [25]. In addition to its catalytic role in quinones, NQO1 has been reported to show superoxide scavenging activity and protective activity against procarcinogenic benzenes [26], [27]. Notably, both *in vivo* and *in vitro* studies have demonstrated that NQO1 regulates the stability of the tumor suppressors p53 and p73, protecting them from 20S proteasomal degradation, which is important for eliminating damaged cells that are prone to cancer development [28], [29], [30], [31]. Therefore, NQO1 is considered an important defense against cancer [25], [31].

**Figure 1:**
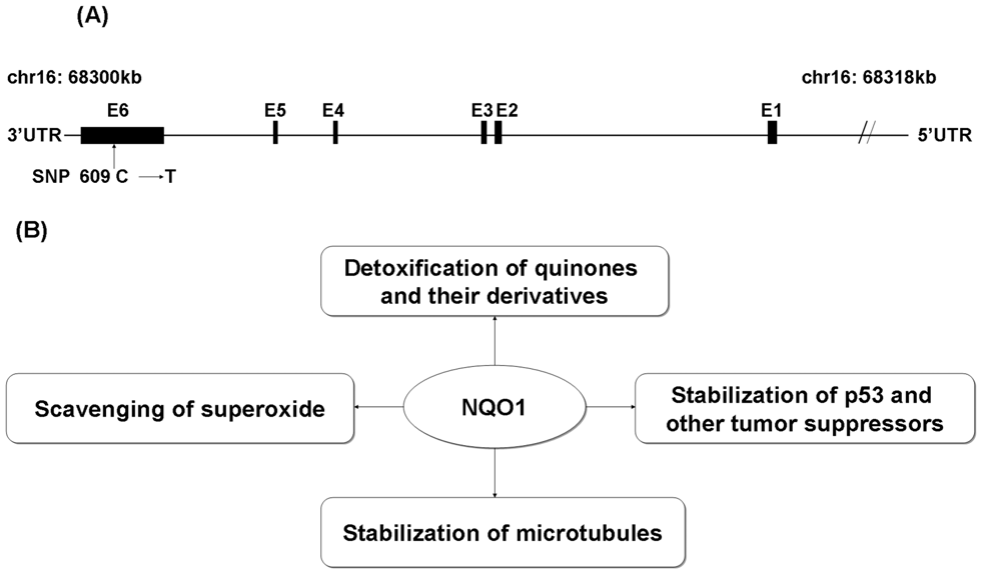
*NQO1* gene structure and its function. A. *NQO1* gene structure and *NQO1* 609C>T polymorphism location. B. The multiple functions of NQO1. As depicted, NQO1 performs multiple functions within the cell, including two-electron reduction of quinones and their derivatives, stabilization of p53 and other tumor suppressors against proteasomal degradation, and scavenging of superoxide. NQO1 has also been implicated in maintaining microtubule integrity.

The *NQO1* gene is located on chromosome 16q22.1, spanning ∼17.2 kb and consisting of 6 exons and 5 introns [32]. To date, there have been 270 SNPs identified in the *NQO1* gene (http://www.ncbi.nlm.nih.gov/SNP). The most extensively studied SNP of *NQO1* is a C-to-T transition at nucleotide position 609 in exon 6 (dbSNP ID: rs1800566, 609C>T; **Figure 1**), which results in a proline-to-serine amino-acid substitution at codon 187 (Pro187Ser) in the protein. Genotype-phenotype studies of the *NQO1* 609C>T polymorphism showed that the variant T allele was associated with reduced NQO1 enzymatic activity in both human cell lines and primary human tissues [24], [33], [34], [35]. Furthermore, there is a clear allele dosage effect of the *NQO1* 609T genotypes on NQO1 enzymatic activity, with the homozygotes (TT) having the lowest, the heterozygotes (CT) having the intermediate, and the wild-type homozygotes (CC) having the highest NQO1 enzyme activity [33], [36], [37], [38]. Decreased NQO1 enzymatic activity is caused by increased polyubiquination and proteosomal degradation of the mutant NQO1 protein [39]. Altered expression of NQO1 protein has been observed in liver, colon, esophagus, stomach, and pancreas cancers [40], [41], [42], [43], [44], [45]. Furthermore, the TT genotype of the *NQO1* 609C>T polymorphism was associated with reduced NQO1 protein expression in tumor tissues from a subset of GI cancer patients (cardiac carcinoma, gastric adenocarcinoma, esophageal adenocarcinoma, and esophageal squamous cell carcinoma) [43], [45]. Because of this SNP's functional consequence, many epidemiological studies have examined the effect of the *NQO1* 609C>T polymorphism on risk of GI cancers, including cancers of the esophagus, stomach, colorectum, pancreas, and liver. However, the reported genetic effects varied across the published studies, and a clear impact of this SNP on cancer risk is also limited by the insufficient statistical power of these individual studies with a relatively small sample size. Therefore, we performed a meta-analysis of published data to evaluate the influence of the *NQO1* 609C>T polymorphism on the risk of GI cancers.

## Materials and Methods

### Identification and eligibility of relevant studies

Using the PubMed search engine, we searched Medline databases, on the association of *NQO1* 609C>T polymorphism (rs1800566) with the risk of GI cancers (defined as cancers of the esophagus, stomach, colorectum, pancreas, gallbladder, liver and small/larger bowel cancer), which had been published up to October 6, 2011 with a limit to human studies in English language. The following keywords were used: ‘NAD(P)H Dehydrogenase (Quinone)’ or ‘NQO1’, ‘polymorphism’, ‘variant’, and in combination with ‘gastrointestinal/aerodigestive tract cancers’, or ‘esophageal cancer’, ‘gastric/stomach’ cancer, ‘colorectal/colon/rectum cancer’, ‘ pancreatic cancer’, ‘liver cancer’, ‘hepatocellular carcinoma’, ‘gallbladder cancer’, and ‘small/larger bowel cancer’. In addition, the references cited in the retrieved studies were also reviewed manually to identify publications on the same cancer type. If studies from the same study group had overlapped subjects, the most recent or largest study was included in the final analysis. Human population-based or hospital-based association studies were included in this meta-analysis, if they met all the following criteria: (1) an independent, unrelated case-control, nested case-control, or cohort study, (2) the *NQO1* 609C>T polymorphism was determined, (3) the outcome was GI cancers, (4) there were sufficient data for calculating an odds ratio (OR) with 95% confidence interval (CI), and (5) the study was reported in English. Exclusion criteria were: (1) duplicate data, (2) abstract, case report, comment, review and editorial, (3) no sufficient genotyping data were provided, (4) the outcome was benign tumors, precancerous lesions, and adenomas, and (5) family-based study.

### Data extraction

Two reviewers (HY and HL) independently reviewed the articles and extracted the data from all eligible publications according to the criteria listed above. The following information was recorded for each study: first author, year of publication, country or region of origin, ethnicity, cancer type, number of cases and controls, number of cases and controls by genotype, source of control group (population-based or hospital-based), genotyping methods, minor allele frequency in controls, method for matching controls to cases. Any discrepancies between the two investigators were resolved by discussion and consultation with a third reviewer (LW).

### Quality score assessment

The quality of included studies was independently assessed by the same two reviewers using the quality assessment criteria, which was modified from previously published meta-analysis of molecular association studies [46], [47]. We included the following factors related to both traditional epidemiological considerations and cancer genetic issues in terms of quality of the studies: representativeness of the cases, representativeness of the controls, ascertainment of GI cancers, control selection, genotyping examination, response rate, and total sample size. The criteria are described in detail in **Table S1,** and the scores were defined as 1 to 3 points given to each component or 0 if absent or the study with a sample size of less than 200. A final quality score was obtained by summation of each component giving a range from 0 (the lowest) to 15 (the highest). Studies scoring<8 were classified as low quality, and those ≥8 as high quality. Disagreements were resolved by consultation with the third reviewer.

### Statistical analysis

Deviation of genotype frequencies of the *NQO1* 609C>T polymorphism in control subjects from Hardy-Weinberg equilibrium (HWE) was tested by using the Chi-square goodness of fit, and a *P* value<0.05 was considered significant. Odds ratio (OR) and corresponding 95% confidence interval (95% CI) were used to estimate the association between the *NQO1* 609C>T polymorphism and cancer risk. We estimated the risk for the variant homozygous TT and heterogeneous CT genotypes, compared with the wild-type homozygous CC genotype, and then for CT/TT *vs.* CC and TT *vs.* CC/CT, assuming both dominant and recessive effect models, respectively. The heterogeneity across studies was assessed with the *Q* test, and the heterogeneity was considered significant when a *P-*value<0.1 for the *Q* statistic [48]. If the heterogeneity was not significant, the fixed-effects model was used to estimate the summary OR and 95% CI; Otherwise, the random-effects model was used [49]. We also calculated the *I* ^2^ index, which can quantify the degree of heterogeneity in a the meta-analysis [50]. The potential source of heterogeneity across studies was explored by stratification and meta-regression analyses. Stratified analyses were conducted by several study characteristics, such as ethnicity, type of cancers (if one cancer type contains less than two studies, it was merged into the ‘other cancers’ group), and quality score of studies (quality score<8 and ≥8). In addition, the studies investigating multiple types of cancers or multiple ethnicities were separated into groups for the subgroup analysis. Both Begg's and Egger's tests [51], [52] were used to test for publication bias. A *P-*value<0.1 was used as an indication for the presence of potential publication bias. Sensitivity analyses were conducted by including and excluding studies not in HWE, and by removing one study at a time to assess the influence of individual studies on the pooled ORs, respectively. All analyses were performed by using Review Manager (v.5.0; Oxford, England) and Stata software (version 8.2; Stata Corp LP, College Station, TX, USA). In addition, for each statistically significant association, we estimated the false positive report probability (FPRP) using the method described by Wacholder et al [53] to evaluate the robustness of the findings. Wacholder et al suggested that estimating statistical power based on the ability to detect an OR of 1.5 (or 0.67 = 1/1.5 for an OR less than 1.0), with an alpha level equal to the observed *P*-value [53]. Because a single nucleotide polymorphism usually shows a relatively small effect size (i.e., OR<1.5), we presented results for an OR of 1.2. An FPRP less than 0.2 was considered as a noteworthy association [53].

## Results

### Characteristics of all included studies

**Figure 2:**
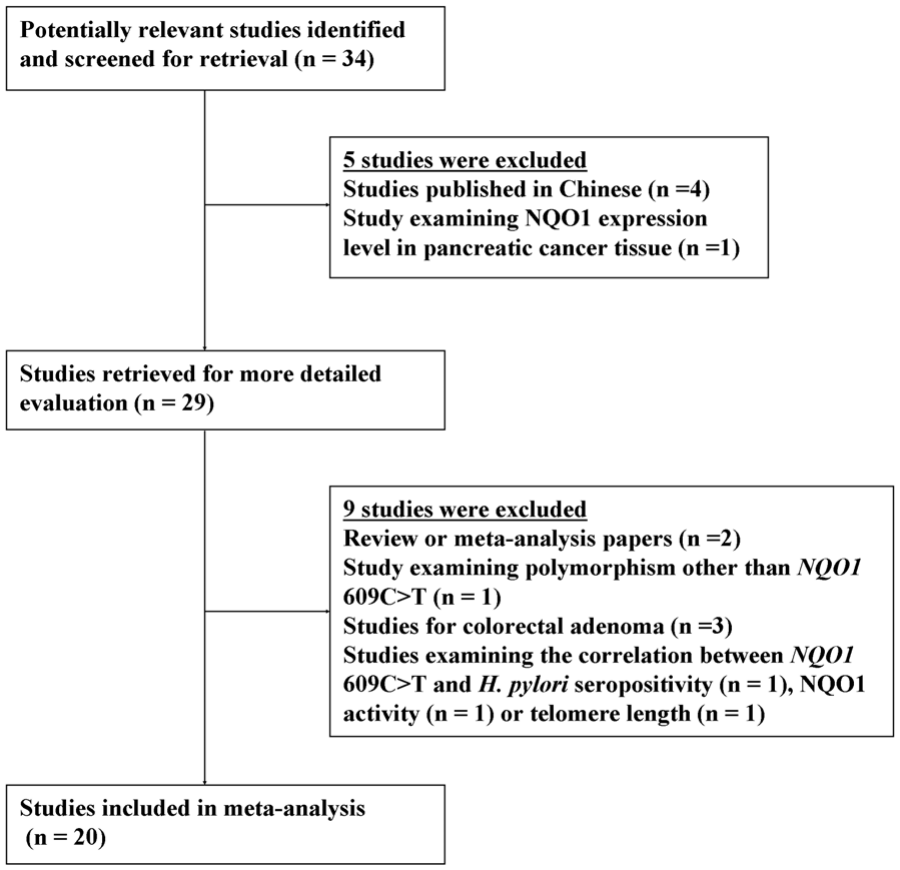
The flow chart of the included studies in the meta-analysis.

As of October 6, 2011, we had identified 29 potentially eligible studies that have investigated the association between the *NQO1* 609C>T polymorphism and risk of GI cancers. After retrieving the full text of these 29 articles, we excluded 9 articles because of the following reasons: one reported the association between the *NQO1* 609C>T SNP and *H. pylori* seropositivity [54]; one did not focus on the *NQO1* 609C>T but on *NQO1* R139W SNP (rs4986998) [55]; three were for the association between the *NQO1* 609C>T SNP and colorectal adenoma [56], [57], [58]; two were for the correlation between the *NQO1* 609C>T genotypes and NQO1 activity [59] or telomere length [60]; two were for review or meta-analysis articles [61], [62]. In addition, the Caucasian control group (252 Caucasian controls) in the study by Zhang et al. [45] had overlapped subjects used in the study by Sarbia et al. [43], and the esophageal cancer patients (193 cases) in the study by Zhang et al. [63] were also overlapped with those in the same author's study [45]. Therefore, these 252 Caucasian controls and 193 esophageal cancer patients were excluded to avoid double counting in our meta-analysis. The flow chart in **Figure 2** summarizes this literature review process.

Overall, data from 20 publications with 5,491 cases and 5,917 controls were available for our meta-analysis. Main characteristics of the included publications are presented in **Table 1**. Among the 20 publications, four studies were for esophageal cancer [45], [64], [65], [66], one for gastric cancer [67], nine for colorectal cancer [55], [59], [68], [69], [70], [71], [72], [73], [74], [75], [76], two for pancreatic cancer [77], [78], one for liver cancer [79], and three for multiple types of GI cancers [43], [63], [80]. Of all studies, 11 studies were conducted in Caucasian populations [43], [65], [66], [68], [71], [72], [73], [74], [75], [76], [77], [78], seven in Asian populations [63], [64], [67], [69], [70], [79], [80], and two in multiple populations [45], [76]. Polymerase chain reaction (PCR)-restriction fragment length polymorphism (RFLP) method was used to determine the genotype in all the included studies except for one by Mohelnikova-Duchonova et al. [78], in which the TaqMan Assay was used. The genotype frequency distributions of the *NQO1* 609C>T polymorphism in controls in 19 of 20 included studies were in agreement with HWE. The HWE test in the study by Lafuente et al. was not mentioned [72]; we also could not perform the HWE test for the subjects (either cases or controls) in that study, because only the total number of the combined genotypes (TT *vs.* CT/CC) was available. Therefore, this study was included in the analysis for the recessive model but not for other genetic models. Quality scores for the individual studies ranged from 4 to 13, with 60.0% (12 of 20) of the studies being classified as high quality (≥8).

**Table 1:** Characteristics of studies included in the meta-analysis.

First author (Reference No.)	Year	Country	Cancer type	Ethnicity	No. of cases/controls	Type of case-control study	Genotyping method	Quality Score	*P*-value^a^
Marjani (64)	2010	Iran	esophagus	Asian	93/50	Hospital-based	PCR-RFLP	5	0.47
Martino (65)	2007	United Kingdom	esophagus	Caucasian	141/93	Hospital-based	PCR-RFLP	5	0.99
Rahden (66)	2004	German	esophagus	Caucasian	140/260	Hospital-based	PCR-RFLP	4	0.17
Sarbia (43)	2003	German	esophagus, stomach, etc	Caucasian	384/252	Hospital-based	PCR-RFLP	6	0.60
Zhang (45)	2003	German	esophagus	Mixed	450/393	Hospital-based	PCR-RFLP	9	0.77
Zhang (63)	2003	China	stomach, esophagus	Asian	124/165	Hospital-based	PCR-RFLP	7	0.39
Hamajima (80)	2002	Japan	esophagus, colorectum, stomach, etc.	Asian	391/640	Hospital-based	PCR-RFLP	7	0.17
Malik (67)	2010	India	stomach	Asian	108/195	Hospital-based	PCR-RFLP	5	0.31
Sachse (75)	2002	United Kingdom	colorectum	Caucasian	490/593	Population-based	PCR-RFLP	11	0.56
Hlavata (68)	2010	Czech	colorectum	Caucasian	495/495	Hospital-based	PCR-RFLP	10	0.85
Sameer (69)	2010	India	colorectum	Asian	86/160	Hospital-based	PCR-RFLP	8	0.45
Nisa (70)	2010	Japan	colorectum	Asian	684/777	Hospital-based	PCR-RFLP	13	0.07
Begleiter (76)	2006	Canada	colorectum	Mixed	280/327	Hospital-based	PCR-RFLP	9	0.29
van der Logt (71)	2006	New Zealand	colorectum	Caucasian	369/415	Population-based	PCR-RFLP	8	0.95
Harth (73)	2000	German	colorectum	Caucasian	323/205	Population-based	PCR-RFLP	9	0.79
Mitrou (57)	2002	United Kingdom	colorectum	Caucasian	206/345	Hospital-based	PCR-RFLP	9	0.96
Lafuente (72)^b^	2000	Spain	colorectum	Caucasian	247/296	Hospital-based	PCR-RFLP	8	-
Mohelnikova-Duchonova (78)	2010	Czech	pancreas	Caucasian	235/265	Hospital-based	TaqMan assay	8	0.80
Bartsch (77)	1998	German	pancreas	Caucasian	81/76	Hospital-based	PCR-RFLP	5	0.27
Akkiz (79)	2010	Turkey	liver	Asian	167/167	Hospital-based	PCR-RFLP	8	0.81

a *P*-value of the chi-square goodness of fit test for Hardy-Weinberg equilibrium (HWE) in controls.

b the HWE test can not be conducted because only the total number of genotypes (TT *vs*. CT/CC) was available, and the HWE test was not mentioned in this study.

### Frequency of the *NQO1* 609 C>T polymorphism in control populations

Of 5,917 control subjects included in this meta-analysis, 3622 were Caucasians and 2295 were Asians. The frequency distributions of the genotypes of the *NQO1* 609 C>T polymorphism were different between these two ethnic groups. The frequencies of the TT, CT, and CC genotypes were 3.1%, 28.2%, and 68.7%, respectively, in Caucasians and 13.1%, 44.7%, and 42.2% in Asians, respectively (**Table 2**).

**Table 2:** The genotype frequencies of the *NQO1* 609C >T polymorphism in controls in different ethnic groups.

Ethnic group	Number of controls	Genotype (%)		
		CC	CT	TT
Caucasians a	3326	2286 (68.7)	937 (28.2)	103 (3.1)
Asians	2295	968 (42.2)	1027 (44.7)	300 (13.1)
*P*-value b		0.007	0.005	0.007

a The study by Lafuente et al was excluded when calculating the genotype frequency because the numbers for the CC and TT genotypes were not provided in this study.

b Two-side Student's *t* test within the stratum.

### Association between the *NQO1* 609C>T polymorphism and the risk of GI cancers

**Figure 3:**
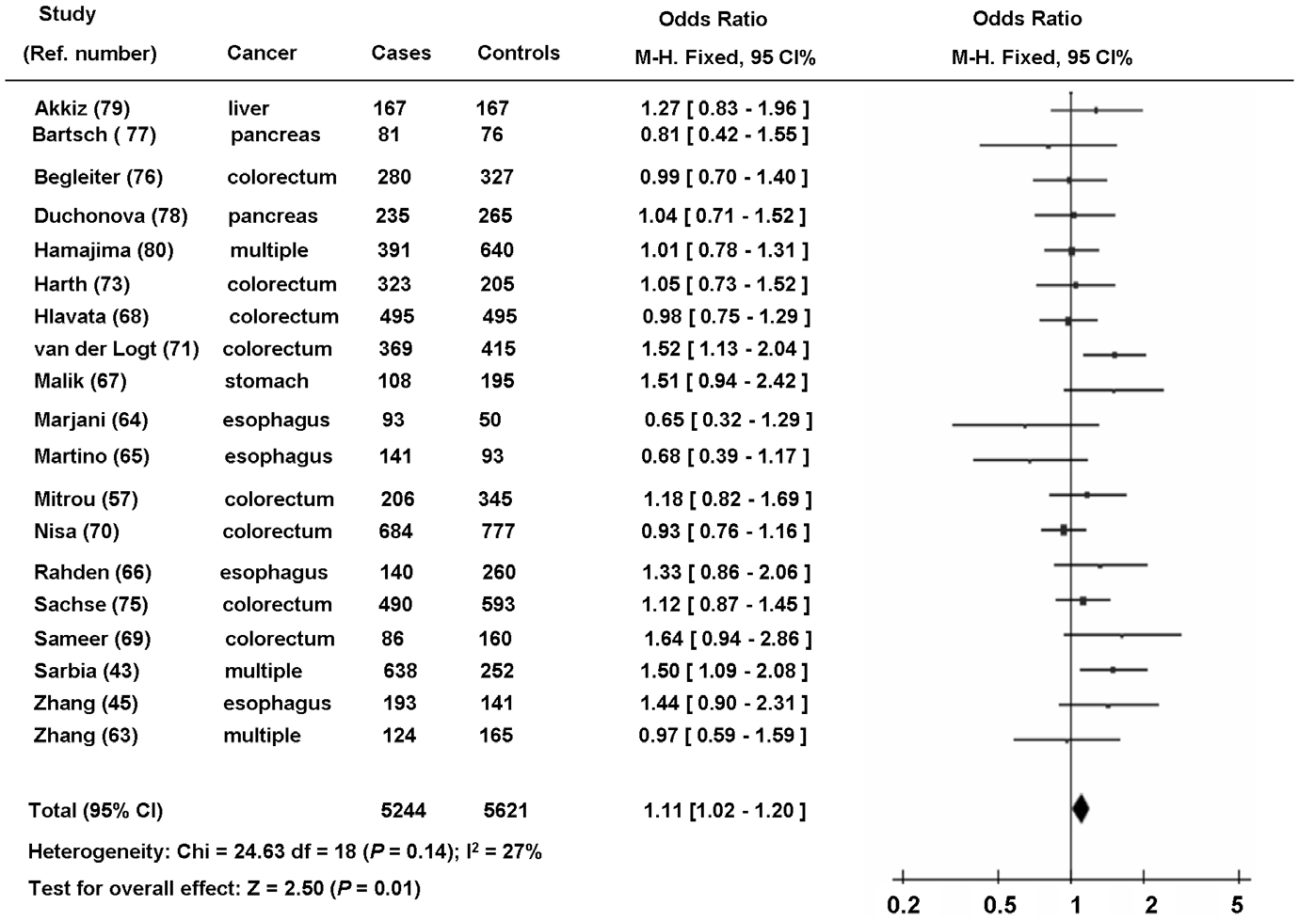
Forest plot (Fixed effects model) describing the association of the *NQO1* 609C>T polymorphism with risk of gastrointestinal (GI) cancers. The *NQO1* 609C>T polymorphism was associated with a modestly increased risk of GI cancers in a dominant model (CT/TT *vs.* CC).

Overall, as shown in **Table 3**, compared to the wild-type CC homozygous genotype, the CT heterozygous genotype was significantly associated with a modestly increased risk for GI cancers (CT *vs.* CC: OR = 1.10, 95% CI = 1.01 – 1.19). A main effect also was significant in the dominant model (CT/TT *vs*. CC: OR = 1.11, 95% CI = 1.02 – 1.20) (**Figure 3**). There was no significant heterogeneity among the studies (*P*_heterogeneity_ = 0.27 and *I^2^* = 0.15 for CT *vs*. CC; *P*_heterogeneity_ = 0.14 and *I^2^* = 0.27 for CT/TT *vs*. CC). We found similar effects in the homozygous comparison (TT *vs*.CC: OR = 1.20, 95% CI: 0.96 – 1.50) and in the recessive model comparison (TT *vs*. CT/CC: OR = 1.22, 95% CI: 0.98 to 1.51). However, these effects did not reach statistical significance. A modest heterogeneity among the studies was observed (*P*_heterogeneity_ = 0.09 and *I^2^* = 0.32 for TT *vs*. CC; *P*_heterogeneity_ *= *0.06 and *I^2^* = 0.36 for TT *vs*. CT/CC). Subsequent sensitivity analyses were performed by removing the individual study sequentially, and we found that all but one Japanese study by Hamajima et al. [80] slightly influenced the overall pooled ORs. After exclusion of this study, a significant increased risk was found in the homozygous comparison (TT *vs*.CC: OR = 1.27, 95% CI: 1.03 – 1.47) or in the recessive model comparison (TT *vs*. CT/CC: OR = 1.29, 95% CI: 1.05 – 1.59), and the heterogeneity among the studies was not significant (*P*_heterogeneity_ = 0.18 and *I^2^* = 0.23 for TT *vs*. CC; *P*_heterogeneity_ *= *0.20 and *I^2^* = 0.20 for TT *vs*. CT/CC), suggesting that this study may contribute to the observed heterogeneity across studies.

**Table 3:** Meta-analysis for the association between the *NQO1* 609C>T polymorphism and cancer risk.

Variables	No. of subjects Cases/Controls	n^a^	CT *vs*. CC			TT *vs*.CC			CT/TT *vs*. CC^b^			TT *vs*. CT/CC^c^		
			OR (95% CI)	*I^2^*	*P* d	OR (95% CI)	*I^2^*	*P* d	OR (95% CI)	*I^2^*	*P* d	OR (95% CI)	*I^2^*	*P* d
Overall	5491/5917	20	**1.10 (1.01 – 1.19)**	0.15	0.27	1.20 (0.96 – 1.50)	0.32	0.09	**1.11 (1.02 – 1.20)**	0.27	0.14	1.22 (0.98 – 1.51)	0.36	0.06
Caucasians	3645/3622	12	**1.13 (1.01 – 1.26)**	0.22	0.24	1.20 (0.91 – 1.58)	0.19	0.27	**1.14 (1.02 – 1.26)**	0.27	0.19	1.25 (0.96 – 1.62)	0.25	0.20
esophagus	599/605	3	1.10 (0.85 – 1.43)	0.20	0.29	1.27 (0.30 – 5.42)	0.76	0.01	1.11 (0.75 – 1.66)	0.59	0.09	1.26 (0.31 – 5.05)	0.75	0.02
stomach	320/252	1	1.62 (1.12 – 2.33)	-	-	2.31 (0.71 – 7.50)	-	-	1.66 (1.16 – 2.37)	-	-	2.00 (0.62 – 6.45)	-	-
colorectum	2410/2676	7	1.13 (0.99 – 1.29)	0.33	0.19	1.11 (0.79 – 1.56)	0.0	0.48	**1.13 (1.00 – 1.28)**	0.11	0.34	1.20 (0.88 – 1.64)	0.27	0.23
pancreas	316/341	2	0.96 (0.68 – 1.35)	0.0	0.46	1.08 (0.49 – 2.37)	0.0	0.88	0.97 (0.70 – 1.35)	0.0	0.52	1.12 (0.51 – 2.43)	0.0	0.97
Asians	1846/2295	8	1.07 (0.94 – 1.23)		0.43	1.25 (0.90 – 1.73)		0.05	1.07 (0.94 – 1.21)		0.17	1.22 (0.90 – 1.67)		0.04
esophagus	388/831	3	1.07 (0.79 – 1.45)	0.23	0.28	1.12 (0.52 – 2.39)	0.62	0.07	1.09 (0.81 – 1.45)	0.43	0.17	1.08 (0.53 – 2.20)	0.64	0.06
stomach	375/1000	3	1.12 (0.85 – 1.47)	0.0	0.20	1.41 (0.98 – 2.03)	0.46	0.22	1.21 (0.93 – 1.56)	0.0	0.12	1.44 (0.84 – 2.46)	0.59	0.24
colorectum	916/1577	3	0.99 (0.82 – 1.18)	0.37	0.20	0.86 (0.65 – 1.13)	0.33	0.22	0.96 (0.81 – 1.15)	0.52	0.12	0.88 (0.68 – 1.14)	0.30	0.24
liver	167/167	1	1.28 (0.82 – 2.00)	-	-	1.24 (0.48 – 3.20)	-	-	1.27 (0.83 – 1.96)	-	-	1.12 (0.44 – 2.83)	-	-
Quality of study														
high	4358/4610	12	**1.10 (1.00 – 1.22)**	0.18	0.27	1.13 (0.92 – 1.39)	0.0	0.48	**1.11 (1.01 – 1.22)**	0.15	0.30	1.17 (0.96 – 1.41)	0.08	0.36
low	1133/1307	8	1.03 (0.86 – 1.24)	0.0	0.45	1.28 (0.77 – 2.13)	0.56	0.03	1.09 (0.92 – 1.30)	0.31	0.18	1.29 (0.79 – 2.10)	0.56	0.03

a Number of studies. The studies investigating multiple types of cancers or multiple ethnicities were separated into groups for the subgroup analysis.

b CT/TT *vs*. CC: Dominant model.

c TT *vs*. CT/CC: Recessive model.

d Heterogeneity across studies.

In the stratified analysis by ethnicity, as shown in **Table 3**, significantly elevated cancer risks were found among Caucasians in the heterozygous genotype comparison (CT *vs*. CC: OR = 1.13, 95% CI: 1.01 –1.26, *P*_heterogeneity_ = 0.24 and and *I^2^* = 0.22) and the dominant model comparison (CT/TT *vs*. CC: OR = 1.14, 95% CI 5 1.02 – 1.26, *P*_heterogeneity_ = 0.26 and *I^2^* = 0.27), but not in the homozygous genotype comparison (TT *vs*. CC: OR = 1.20, 95% CI: 0.91 – 1.58, *P*_heterogeneity_ = 0.27 and *I^2^* = 0.19) and the recessive model comparison (TT *vs*. CT/CC: OR = 1.25, 95% CI: 0.96 – 1.62, *P*_heterogeneity_ = 0.20 and *I^2^* = 0.25). No significant heterogeneity was observed for all the genetic mode1 comparisons. The leave-one-out sensitivity analysis found that no single study dramatically influenced the overall pooled ORs (data not shown). In Asians, no significant association between the *NQO1* 609C>T polymorphism and the risk of GI cancers was found for all variant genotypes (CT *vs*.CC: OR = 1.07, 95% CI: 0.94 – 1.23, *P*_heterogeneity_ = 0.43 and *I^2^* = 0.0; TT *vs*.CC: OR = 1.25, 95% CI: 0.90 – 1.73, *P*_heterogeneity_ = 0.05 and *I^2^* = 0.50), the dominant model (CT/TT *vs*. CC: OR = 1.07, 95% CI: 0.94 – 1.26, *P*_heterogeneity_ = 0.17 and *I^2^* = 0.32) and the recessive model (TT *vs*. CT/CC: OR = 1.22, 95% CI: 0.90 – 1.21, *P*_heterogeneity_ = 0.27 and *I^2^* = 0.52). However, the leave-one-out sensitivity analysis showed that after removing the study by Hamajima et al. [80], the heterogeneity among studies diminished, and a significant association was found in the recessive model (TT *vs*.CT/CC: OR = 1.36, 95% CI: 1.02 – 1.81, *P*_heterogeneity_ = 0.23 and *I^2^* = 0.26). In further stratification analysis by cancer site (**Table 3**), a modestly significant increased risk was found for the colorectal cancer under the dominant model in Caucasians (CT/TT *vs*. CC: OR = 1.13, 95% CI: 1.00 – 1.28, *P*_heterogeneity_ = 0.34 and *I^2^* = 0.11). However, no significant association was observed for other cancer sites either in Caucasians or in Asians. The leave-one-out sensitivity analysis showed that no single study dramatically influenced the overall pooled ORs (data not shown).

We also performed subgroup analysis by quality score of studies (**Table 3**). We found that the CT heterozygous genotype was significantly associated with a modestly increased risk for GI cancers, compared to the wild-type homozygous genotype (CC) in the studies with high quality score (≥8.0) (CT *vs*.CC: OR = 1.10, 95% CI: 1.00 – 1.22; *P*_heterogeneity_ = 0.27 and *I^2^* = 0.18), and such an effect was also found in the dominant genetic model (CT/TT *vs*.CC: OR = 1.11, 95% CI: 1.01 – 1.22; *P*_heterogeneity_ = 0.30 and *I^2^* = 0.15). Similar effects were also found for the homozygous genotype comparison (TT *vs*.CC: OR = 1.13, 95% CI: 0.92 – 1.39; *P*_heterogeneity_ = 0.48 and *I^2^* = 0.0) and for the recessive genetic model comparison (TT *vs*.CT/CC: OR = 1.17, 95% CI: 0.96 – 1.41; *P*_heterogeneity_ = 0.36 and *I^2^* = 0.08), though they did not reach statistical significance. In the subgroup of low quality studies, no significant association between the *NQO1* 609C>T polymorphism and the risk of GI cancers was observed. Sensitivity analyses showed that no single study influenced quantitatively the overall pooled ORs (data not shown).

### Evaluation of heterogeneity

In the present study, we used the *Q* test and the *I^2^* index to evaluate the heterogeneity across studies. As shown in Table 2, although the *Q* test showed that there was no significant heterogeneity in some overall comparisons and subgroup analyses, the *I^2^* index suggested that a low to high heterogeneity across studies presented in most of comparisons. We assessed heterogeneity across studies by ethnicity, cancer site, and quality of studies, and found that they did not contribute the heterogeneity observed across the studies in the overall meta-analysis (TT *vs*.CC: t = −0.24, *P* = 0.815 for ethnicity, t = 0.02, *P* = 0.988 for cancer sites, and t = 0.39 8, *P* = 0.703 for quality of studies; TT *vs*.CT/CC: t = 0.00, *P* = 1.000 for ethnicity, t = −0.29, *P* = 0.773 for cancer sites, and t = 0.29, *P* = 0.777 for quality of studies). These factors were also not found to contribute to the heterogeneity across studies in some of the subgroup analysis (data not shown). Together with the results from the leave-one-out sensitivity analysis as mentioned above, the study by Hamajima et al. could be the main source of the observed heterogeneity across the studies in this meta-analysis.

### Publication bias

**Figure 4:**
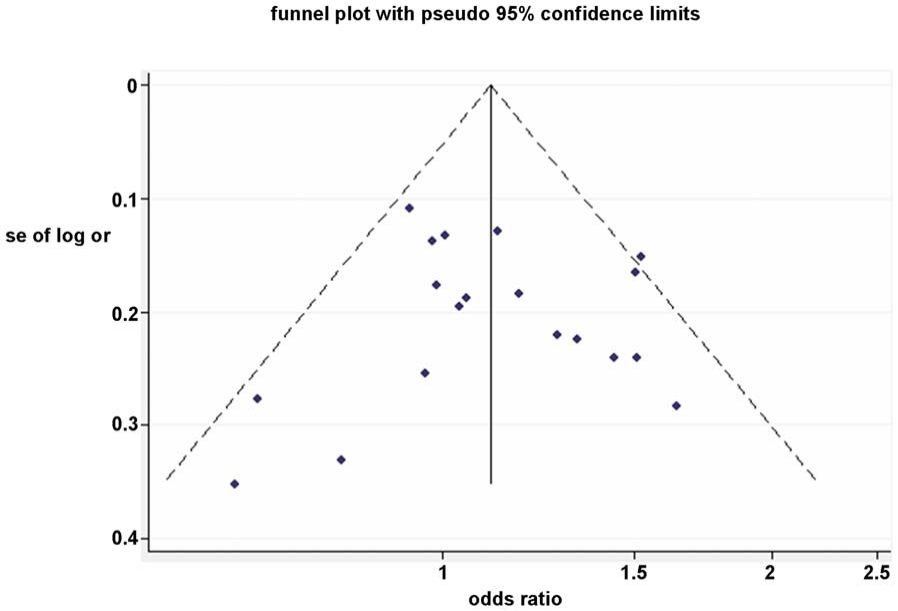
Funnel plot analysis to detect publication bias. Each point represents an individual study for the indicated association.

Both Begg's and Egger's tests were performed to evaluate the publication bias of the included studies. The shape of the funnel plots did not reveal any evidence of obvious asymmetry for all genetic models in the overall meta-analysis (**Figure 4**). The Begg's test and Egger's test did not present any significantly statistical evidence of publication bias for any of the genetic models (CT *vs*.CC: *P*_Begg_ *= *0.529 and *P*_Egger_ *= *0.369, TT *vs*.CC: *P*_Begg_ *= *0.726 and *P*_Egger_ *= *0.690, CT/TT *vs*.CC: *P*_Begg_ *= *1.000 and *P*_Egger_ *= *0.671, and TT *vs*.CT/CC: *P*_Begg_ *= *0.626 and *P*_Egger_ *= *0.700.) Neither funnel plots nor Begg's and Egger's tests detected any obvious evidence of publication bias in the subgroup analyses for all genetic models (data not shown).

Finally, because many subgroup comparisons were conducted, we calculated false positive report probability (FPRP) for each statistically significant result. As shown in **Table 4**, with the assumption of a moderate prior probability of 0.1 and the OR for the specific genotype was 1.2, the FPRP values for the significant findings in the heterozygous genotype comparison (CT vs. CC) and the dominant model (CT/TT vs. CC) in all subjects, and in the dominant model in Caucasians (CT/TT vs. CC) were 0.138, 0.074, 0.099, respectively. However, greater FPRP values were observed for other significant associations between the *NQO1* 609C>T polymorphism and risk of GI cancers.

**Table 4:** False positive reporting probability values for associations between the *NQO1* 609C>T polymorphism and the risk of GI cancers.

Genotype	OR (95% CI)	Prior probability				
		0.25	0.1	0.01	0.001	0.0001
All subjects						
CT *vs*. CC	1.10 (1.01 – 1.19)	0.051	0.138	0.638	0.947	0.994
CT/TT *vs*. CC	1.11 (1.02 – 1.20)	0.026	0.074	0.469	0.899	0.989
Caucasians						
CT *vs*. CC	1.13 (1.01 – 1.26)	0.088	0.225	0.762	0.970	0.997
CT/TT *vs*. CC	1.14 (1.02 – 1.26)	0.035	0.099	0.547	0.924	0.992
Colorectum cancer in Caucasians						
CT/TT *vs*. CC	1.13 (1.00 – 1.28)	0.165	0.373	0.867	0.985	0.998
High quality of study						
CT *vs*. CC	1.10 (1.00 – 1.22)	0.184	0.403	0.881	0.987	0.999
CT/TT *vs*. CC	1.11 (1.01 – 1.22)	0.088	0.224	0.761	0.970	0.997

## Discussion

In the present meta-analysis with 5,491 cases and 5,917 controls, the variant CT heterozygous genotype and the combined CT/TT genotype of the *NQO1* 609 C>T polymorphism was found to be associated with a modestly increased risk of GI cancers, and no significant heterogeneity was found across studies. It was also noted that, when limiting the pooled analysis to the studies with high quality, the results were persistent and robust, with the *NQO1* 609 T allele being significantly associated with the increased risk of GI cancers. Publication bias was not observed in this study. These findings suggest that the *NQO1* 609C>T polymorphism may modify the risk of GI cancers.

Our findings have some biological plausibility, because NQO1 performs multiple functions within the cell. Conclusive evidence suggests that NQO1 has a protective function in cellular defense against the toxicity of electrophilic and oxidizing metabolites of xenobiotic quinones [81]. In addition, its induction protects cells against carcinogenesis [27], [28], [29], [30], [31], [81]. Constitutive expression of NQO1 has been found in most human tissues, where its expression is highly induced by various stimuli, including antioxidants, oxidants, xenobiotics, heavy metals, UV light, and ionizing radiation [37]. It has been shown that NQO1 is overexpressed in many human tumors, including cancers of the lung, breasts, liver, esophagus, stomach, colon, pancreas, and bladder [24], [31], [42], [43], [82], [83], [84], [85]. The *NQO1* knockout mice were reported to exhibit marked increased sensitivity to 7,12-dimethylbenz(a)anthracene (DMBA)- and benzo(a)pyrene (BP)-induced skin carcinogenesis [86], [87].

Human *NQO1* is polymorphic [88], of which the *NQO1* 609C>T polymorphism, in terms of its frequency and phenotypic consequences, is most prominent and thus intensively studied. Our results are consistent with the potentially altered biological functions of NQO1 by the 609C>T polymorphism. Although the association of the homozygous variant genotype (TT) with overall cancer risk did not achieve statistical significance, the magnitude and direction for association for GI cancers were persistent in both overall and some subgroups in our meta-analysis. Because the frequency of the TT genotype of the *NQO1* 609C>T polymorphism was low in the published study populations, with 3.1% and 13.1% of the controls being the TT homozygote in Caucasians and Asians, respectively, we might not have sufficient statistical power to detect the weak effect of this variant genotype on risk of GI cancers. Further studies with larger sample sizes are warranted.

GI cancers represent a heterogeneous group of malignancies. Except for some shared risk factors, different primary sites of GI cancers have different risk factors and thus different etiologies. For example, in addition to smoking and alcohol consumption, *H. Pylori* infection is involved in stomach cancer and HBV/HCV infection is involved in liver cancer, while dietary exposure to heterocyclic amines (HCAs), nitrosamines, polycyclic aromatic hydrocarbons (PAHs) derived from red meat and processed meat is a key risk factor for colorectum cancer. Such etiologic heterogeneity in GI cancers raises the possibility that the *NQO1* polymorphism may be associated with specific types of GI cancers, because NQO1 plays an important role in detoxifying dietary carcinogenic compounds such as HCAs, PAHs and nitrosamines [89]. Therefore, the functional *NQO1* 609 C>T polymorphism resulting in decreased activity of NQO1 enzyme may increase risk of colorectum cancer. Indeed, in the stratification analysis by cancer site in Caucasians and Asians, significantly elevated risk associated with the *NQO1* 609T allele was only found for colorectal cancer among Caucasians but not in Asians. A previous meta-analysis by Chao et al. [62] found an association between the *NQO1* 609T allele and an increased risk for colorectal cancer (CT/TT *vs.* CC: OR = 1.18, 95% CI: 1.02 – 1.35) among 1637 cases and 1854 controls in the Caucasian population, and this association remained statistically significant in this expanded meta-analysis that had included additional subjects (2410 cases and 2676 controls in the Caucasian population). However, the meta-analysis for the *NQO1* 609C>T polymorphism and colorectal cancer risk was not performed in Asian population in Chao's study. We also did not find significant associations between this SNP and risk of other cancer sites, such as cancers of the esophagus, stomach, and pancreas either in Caucasians or in Asians. This lack of significance could be due to either no effect of this SNP on these cancer sites or limited statistical power to detect such a weak association. In our meta-analysis, only two studies with 316 cases and 341 controls for pancreatic cancer in Caucasians, three studies with 375 cases and 1,000 controls for gastric cancer and three studies with 916 cases and 1577 controls for colorectum were conducted in Asians. Therefore, our results should be interpreted with caution. Because the *NQO1* 609C>T polymorphism is functional and potentially to be associated with risk of cancer as shown in this meta-analysis, further larger studies are needed, especially for non-colorectal GI cancers in Caucasians and GI cancers in Asians.

Certain potential limitations exist in our meta-analysis. Firstly, although the Begg's test and Egger's test did not show any publication bias, selection bias could have occurred, because only studies published in English were included in our meta-analysis. Secondly, all the studies included in this meta-analysis were hospital-based case-control studies. In this instance, the hospital-based controls may not be representative of the general population. Thirdly, the numbers of published studies were still not sufficiently large for the analysis of the effect of the variant TT genotype on risk of GI cancers and for some subgroups. Furthermore, we were unable to perform further subgroup analyses for a particular cancer site in different ethnic populations due to a limited number of published studies available to be included. For example, only one Caucasian study and one Asian study for gastric cancer and liver cancer were available for this meta-analysis, respectively. Fourthly, the FPRP analyses showed that with the assumption of a prior probability of 0.1, the FPRP values for the significant findings in overall comparisons and the comparison in the dominant model in Caucasians were below 0.2, providing some measures of robustness for our observations. However, greater FPRP values were observed for the other significant associations between the *NQO1* 609C>T polymorphism and risk of GI cancers, suggesting some possible bias in the findings. Finally, due to lacking individual original data, we did not take into account the other factors such as sex, ethnicity, smoking and drinking status, that could modify the risk of estimate [57], [62], [69], when we evaluated the effect of the *NQO1* 609 C>T polymorphism on the risk of GI cancers. A more precise analysis could have been conducted, if individual data were available. Furthermore, gene-environment and gene-gene interactions should also be considered in further studies.

In summary, despite the above-mentioned limitations, our meta-analysis suggests that the minor allele T of the *NQO1* 609C>T polymorphism may be associated with a moderately increased risk of GI cancers. Although the effect on cancer risk may be modified by ethnicity and cancer sites, small sample sizes in some subgroups suggest that future large and well-designed studies in different ethnic populations and different sites of GI cancers are needed to validate our findings.

## Supporting Information

Table S1 Score of quality assessment.
(DOC)
