# Supplementary material for: A Functional NQO1 609C>T Polymorphism and Risk of Gastrointestinal Cancers: A Meta-Analysis
Source: PLoS One. 2012 Jan 17;7(1):e30566. doi: 10.1371/journal.pone.0030566 (PMC3260285; doi:10.1371/journal.pone.0030566)
Supplement: Table S1 — Score of quality assessment. (DOC) [file pone.0030566.s001.doc]

| **Table S1. Score of quality assessment** | |
| --- | --- |
| **Criteria** | **Score** |
| Representativeness of case |  |
| Selected from population cancer registry | 2 |
| Selected from hospital | 1 |
| No method of selection described | 0 |
| Representativeness of control |  |
| Population-based | 3 |
| Blood donors | 2 |
| Hospital-based (cancer-free patients) | 1 |
| Not described | 0 |
| Ascertainment of gastrointestinal cancer |  |
| Histopathologic confirmation | 2 |
| by patient medical record | 1 |
| Not described | 0 |
| Control selection |  |
| Controls matched with cases by age and sex | 2 |
| Controls matched with cases only by age or by sex | 1 |
| Not matched or not descried | 0 |
| Genotyping examination |  |
| Genotyping done under blinded condition |  |
| Unblinded or not mentioned |  |
| Response rate |  |
| Response rates for both groups within 10% | 2 |
| Response rates are more than 10% | 1 |
| Not mentioned | 0 |
| Total sample size |  |
| Larger than 1000 | 3 |
| Larger than 500, but less than 1000 | 2 |
| Larger than 200, but less than 500 | 1 |
| Less than 200 | 0 |
